# The Jenner Society

**Published:** 1898-11-12

**Authors:** 


### Editor's Letter-Box.

[Our Correspondents are reminded that prolixity is a great bar to publication, and that brevity of style and conciseness of statement greatly facilitate early insertion.]

### THE JENNER SOCIETY.

Dr. Francis T. Bond writes: Whilst thanking you for the sympathetic notice of the Jenner Society in your last issue, I should like to be permitted to correct a misconception which it contains. You say that "till lately the Jenner Society has been not only a modest but a silent one." I trust that it will not diminish the claim of the society to the former characteristic to say that it has been far from silent. During the year 1897, as appears from the annual report—a copy of which I enclose with this—the committee procured the insertion of more than a hundred letters and articles in the London and provincial press in reply to misrepresentations of anti-vaccinators. This year the number of such communications has been more than doubled. In addition, the society has a large personal correspondence, and is constantly being applied to for information and assistance by those who are anxious to meet anti-vaccination misrepresentations, and it has circulated a large amount of pro-vaccination literature. It is, therefore, scarcely fair to say that it has been "silent." I venture to say that it has been as much in evidence in the public press and in other ways as the resources at its disposal allowed. It is, indeed, deeply indebted to the leading journals of the country, so many of which have, as The Hospital has done, commended its objects and advocated support for it. But it takes time and work to get such a movement as this generally recognised, and to enable it to struggle successfully for existence amongst the multitude of claimants for help from the public.

*** Dr. Bond's quotation is inaccurate, and we certainly never suggested that the Jenner Society had been silent during the years he mentions.—Ed. *T. H.*

